# Application of Crop Growth Models to Assist Breeding for Intercropping: Opportunities and Challenges

**DOI:** 10.3389/fpls.2022.720486

**Published:** 2022-02-04

**Authors:** Martin Weih, Eveline Adam, Giulia Vico, Diego Rubiales

**Affiliations:** ^1^Department of Crop Production Ecology, Swedish University of Agricultural Sciences (SLU), Uppsala, Sweden; ^2^Saatzucht Gleisdorf GmbH, Gleisdorf, Austria; ^3^Institute for Sustainable Agriculture, Consejo Superior de Investigaciones Científicas (CSIC), Córdoba, Spain

**Keywords:** APSIM, biodiversity, complementary resource use, plant breeding, process-based models, STICS, mixed cropping

## Abstract

Intercropping of two or more species on the same piece of land can enhance biodiversity and resource use efficiency in agriculture. Traditionally, intercropping systems have been developed and improved by empirical methods within a specific local context. To support the development of promising intercropping systems, the individual species that are part of an intercrop can be subjected to breeding. Breeding for intercropping aims at resource foraging traits of the admixed species to maximize niche complementarity, niche facilitation, and intercrop performance. The breeding process can be facilitated by modeling tools that simulate the outcome of the combination of different species’ (or genotypes’) traits for growth and yield development, reducing the need of extensive field testing. Here, we revisit the challenges associated with breeding for intercropping, and give an outlook on applying crop growth models to assist breeding for intercropping. We conclude that crop growth models can assist breeding for intercropping, provided that (i) they incorporate the relevant plant features and mechanisms driving interspecific plant–plant interactions; (ii) they are based on model parameters that are closely linked to the traits that breeders would select for; and (iii) model calibration and validation is done with field data measured in intercrops. Minimalist crop growth models are more likely to incorporate the above elements than comprehensive but parameter-intensive crop growth models. Their lower complexity and reduced parameter requirement facilitate the exploration of mechanisms at play and fulfil the model requirements for calibration of the appropriate crop growth models.

## Introduction

Intercropping is the simultaneous cultivation of at least two crops in the same field (Willey, 1979), although without necessarily sowing or harvesting them at the same time. Intercropping has been a common agricultural practice over ages; however, the intensified agriculture of the last decades is based on uniform crops relying on mechanization and heavy use of synthetic fertilizers and pesticides, which has reduced intercropping (Hauggaard-Nielsen and Jensen, 2001). The negative side impacts of intensive agriculture on soil, water, and air quality and on biodiversity conservation are calling for a renewed interest on intercropping, among other practices (Malezieux et al., 2009).

Intercrops often use available resources more efficiently than the corresponding sole crops, as intercropped species can utilize resources in a complementary way and take advantage of other mechanisms such as niche facilitation. However, the outcome and success of intercrops depends on the competitive hierarchies and the role of asymmetric competition among the admixed species, as well as their individual performances (Andersen et al., 2007). For example, in cereal-legume intercrops, the cereal component is often a better competitor for soil inorganic nitrogen (N) than the legume component especially in early growth stages, due to rapid and deep root growth; while the legume component can exploit fixed *N* mainly in later crop growth stages when the soil N availability increasingly limits crop growth (Bedoussac et al., 2015). The mechanisms underlying competitive hierarchies and positive interactions can include the complementary use of less mobile soil resources such as phosphorus (Hinsinger et al., 2011), with several legume species being able to facilitate the acquisition of phosphorus by associated cereals (Li et al., 2014).

Traditionally, intercropping systems have been developed and improved by empirical methods within a specific local context, e.g., by combining species and varieties with anticipated complementary resource use and niche differentiation in a certain region. To support the development of promising intercropping systems, the individual component species of mixtures can be subject of breeding and genotypes with contrasting resource foraging characteristics selected to maximize mixture complementarity, reduce negative competitive interactions, and improve the production of each component species (Litrico and Violle, 2015). Such a process can be facilitated by modeling tools simulating, in a system approach, plant functioning and expected outcomes of the combination of different species’ (or genotypes’) traits for growth and yield development over time. As such, models can support the exploration of a wide range of plant properties and growth conditions without the need of extensive and time-consuming field testing; and even before actually breeding for these plant properties. Crop models have already been used successfully to assist plant breeding (Rötter et al., 2015). Yet, the focus of the previous approaches was on the design of cultivars grown as sole crops. Modeling intercrops involves additional challenges, mainly because often complex plant–plant interactions need to be considered; although, the underlying mechanisms in many of them still are poorly known. We discuss current advances and future directions for a more effective use of models in support of breeding for intercropping. We do this by (i) summarizing the specific challenges associated with breeding for intercropping; (ii) providing an update on existing crop growth models that can simulate intercrops; and (iii) evaluating their application to assist breeding for intercropping.

## Challenges in Breeding for Intercropping

Breeding would be a straightforward task if the desired traits were clear, there was genetic variation in the traits to be selected for, and accurate but fast and economic screening protocols were available for the massive screenings needed. Breeding success can be accelerated by adoption of valuable emerging technologies for phenotyping and genotyping. However, proper identification, prioritization of the traits and their combinations to target among the many possible ones remains a major challenge. Their definition is needed before they can be selected individually or in combinations in large segregating populations following different selection strategies (Annicchiarico et al., 2019; Bancic et al., 2021; Wolfe et al., 2021). In the absence of such knowledge on traits and trait combinations, breeding is still possible but needs to rely on heavy experimentation using proper designs (Barot et al., 2017; Haug et al., 2021). A better understanding of the mechanisms underlying intercropping benefits would facilitate the search of the existing variation for the traits of interest and enhance the breeding success. To date, the bottleneck remains our understanding of the most relevant traits to breed for in an intercrop.

In general, trait selection and crop breeding can be performed either on sole crops or intercrops. Yet, selection efficiency for intercropping adaptation under sole cropping conditions is generally moderate or low (Annicchiarico et al., 2019), and elite cultivars selected for sole cropping systems might not be the optimal ones for intercropping. This is because, in a sole crop, desirable traits are often those increasing resource acquisition, whereas, when the same crop is grown in an intercrop, traits that optimize complementarity or facilitation can be more relevant (Costanzo and Bàrberi, 2014) but require considering complex above- and below-ground interspecific interactions. Also, traits are plastic and likely differ when plants are grown in sole crops or intercrops; and the often observed significant genotype × cropping system interactions indicate that specific breeding for intercropping is needed to exploit the genetic variability of the traits of interest in an intercrop context (Nelson and Robichaux, 2006).

Particularly important, in an intercrop context, are traits related to competitive ability and compatibility, which can be selected for by incorporating the relevant traits into selection indices (Annicchiarico, 2003; Annicchiarico and Filippi, 2007). Still, the relevant adaptive traits can vary with the intercropping systems and over time, reinforcing the need for careful considerations of appropriate trait combinations (Jensen et al., 2015). For example, in general, leaf area, leaf area development, and plant height are all expected to enhance competitive ability. In a specific case, pea competitive ability was affected mainly by leaf area in early growth stages and plant height later on (Barillot et al., 2014), which needs to be considered when this species is to be grown in an intercrop. While leafless pea types are desired in sole crops to improve standing ability, leafy types might be preferred in intercrops due to a higher growth rate and competitive ability (Semere and Froud-Williams, 2001). Thus, breeding for intercrops requires setting specific objectives for each of the admixed species in relation to the other(s). For example, in cereal-legume mixtures the legume component is often less competitive due to a lower relative growth rate, so we could admix less competitive cereals or try to improve the competitiveness of the legume. This can be achieved by selecting for (i) higher relative growth rate and plant height in the legume or lower in the cereal or both; (ii) greater plasticity of both, with implications for plant competition for light, including higher light absorption capacity under shading (Wang et al., 2006); and (iii) early establishment of rhizobium symbiosis in legumes (Hauggaard-Nielsen and Jensen, 2001).

The challenge is to discern the most relevant traits contributing to the possible intercropping benefits, and to prioritize them according to their predicted breeding value. Modeling could help to understand the net outcome of the complex interactions among the components’ traits affecting complementarity and facilitation in intercrops; and to define how specific traits should be changed to take maximal advantage of complementarity and other intercropping benefits. In principle, there are two types of models, i.e., process-based and empirical models. Process-based models simulate detailed physical or biological processes inherent a system, while empirical models rely on correlative relationships in line with mechanistic understanding, but without fully describing the inherent processes. In reality, most models use a hybrid approach and combine process-based and empirical elements. Empirical approaches involve great uncertainty and bias especially when correlative relationships are extrapolated beyond observed variability. Simulation of the processes behind plant–plant and plant–environment interactions in intercrops usually involves the extrapolation of relationships beyond observed variability, because most of the available data sets are from sole crops and represent the relevant relationships under past conditions which not necessarily are the same in future conditions. The best suited models to address the complex interactions in intercrops are therefore those that explicitly describe the processes behind plant–plant and plant–environment interactions, as reviewed next.

## Process-Based Models to Simulate Intercrops

Mathematical process-based crop growth models integrate plant properties and environmental conditions in a system approach. They simulate plant functioning based on the individual plant properties of crop species or cultivars and the environmental and management (e.g., intercropping) conditions at the target location. Crop growth models quantify the final outcome of these interacting aspects on, e.g., crop yields, without depending on lengthy field test campaigns. As such, they can assist plant breeding, by highlighting which plant properties are sensitive to the model simulation conditions, how the outcome of these properties would respond to the anticipated changes in growth conditions, and ultimately where significant performance gains can be made by breeding.

Despite their promises, hitherto only few crop models have been developed and applied to simulate intercrops. Simple models have been developed to evaluate how plant–plant interactions in terms of competition and facilitation can affect plant growth and seed yield (Tilman et al., 1997; Klimek-Kopyra et al., 2013; Evers et al., 2019), but these models cannot be used to predict the net outcome of species mixtures in agriculture. Among the models designed for agronomic applications, the most frequently used for intercrops are APSIM (Agricultural Production Systems sIMulator) (Keating et al., 2003; Knörzer et al., 2011a; Chimonyo et al., 2016; Berghuijs et al., 2021) and STICS (Simulateur mulTIdisciplinaire pour les Cultures Standard, or multidisciplinary simulator for standard crops) (Brisson et al., 2003, 2004; Corre-Hellou et al., 2009). Other crop growth models able to simulate intercrops are Daisy (Manevski et al., 2015) and DSSAT-CERES (Knörzer et al., 2011b; for reviews of model applications to intercrops, see Knörzer et al., 2010 and Chimonyo et al., 2015). Simulating intercrops with these models is often challenging, because they use many parameters, for which measured values from field experiments are required as inputs. Given the limited set of parameters typically available from most field experiments using intercrops, the uncertainties in the estimates of these parameters are large, and consequently the resulting simulation results are uncertain. An alternative approach is that of minimalist crop growth models. These models rely on fewer parameters, thus reducing the uncertainties in parameter estimations. These models also facilitate model adjustment to various species or variety combinations grown in an intercrop under different conditions (Van der Werf et al., 2007). Minimalist crop models have been recently developed for strip intercrops of wheat and maize (Gou et al., 2017; Liu et al., 2017; Tan et al., 2020) or wheat and faba bean grown under nitrogen-limited conditions (Berghuijs et al., 2020).

Modeling an intercrop requires capturing the extent of interspecific competition for limiting resources and how that is determined by the properties of the admixed species. In this context, it is interesting to note that process-based growth models often depart from conditions of unlimited plant growth. The simulated potential plant growth is then reduced by the environmental factors considered relevant, including neighbors of different species competing for the same resources. For example, the competitive ability of the species involved was affected by differences in canopy structure and crop height (Keating and Carberry, 1993; Pronk et al., 2003; Gou et al., 2017), root system architecture (Ozier-Lafontaine et al., 1998; Corre-Hellou et al., 2007), and nutrient uptake capacity (Corre-Hellou et al., 2006). The APSIM model considers these effects *via* the following crop-related model parameters: phenology stage (usually defined in degree days), leaf development and biomass growth rates, radiation use efficiency (RUE) in g biomass per unit of light, and water and/or nitrogen demand and deficit functions (Chimonyo et al., 2016; Berghuijs et al., 2021). The STICS model provides additional examples for crop related parameters that can account for the competitive ability and how it changes in intercrops: minimum and maximum root and biomass growth rates and species specific nitrogen dilution functions derived from theoretical optimum nitrogen contents in the admixed target species (Brisson et al., 2003; Corre-Hellou et al., 2009). Finally, in minimalist models, the following crop related parameters describing inter-specific competition have been used: minimum and maximum plant heights, relative growth rate, specific leaf area, RUE, nitrogen demand and dilution functions (Gou et al., 2017; Berghuijs et al., 2020; Tan et al., 2020).

## Discussion

Crop growth models use plant parameters to simulate growth and development of a crop for the given environmental conditions. These models have been used previously to assist plant breeding, especially ideotype breeding of crops to be grown in sole culture (Martre et al., 2015; Rötter et al., 2015). In contrast to the many applications for growth models simulating sole crops, few crop growth models have been developed, calibrated, and validated for intercrops, to accommodate the specific mechanisms of plant–plant interactions that are important in intercrop performance (Knörzer et al., 2010; Chimonyo et al., 2015).

A remaining challenge for modeling intercrops is including the most important mechanisms of plant–plant interactions, such as different kinds of cues for neighbor detection: light quality (initiating, e.g., shading adaptation), root chemicals, and volatile organic compounds (Biedrzycki et al., 2010; Gruntman et al., 2017; Ninkovic et al., 2019). Most existing models applicable to intercrops lack several of these mechanisms. For example, APSIM does not simulate shading adaptation of the shorter species in the intercrop, and therefore systematically overestimates the growth of the taller species and underestimates the performance of the shorter species (Berghuijs et al., 2021). However, the addition of plant characteristics and mechanisms driving interspecific competition to existing crop growth models, such as APSIM and STICS, would make these already complex models even more so. There would be a further increase in the number of plant and environment parameters, which then would need to be assessed several times during a single growing season for model calibration. Among them, some are not commonly or easily monitored in the field trials targeted to plant breeding.

Yet, the most important limitation in using crop growth models in support of breeding for intercrops is the difficulty to link model parameters to breeding traits. While the parameter lists for many crop growth models include some “true traits”—i.e., plant characteristics that breeders could select for—many of the parameters included in these models cannot be easily translated into breeding traits. Hence, in spite of the strength of crop growth models in identifying highly influential plant properties, many of these properties are likely to be driven by the expression of several underlying traits and are, therefore, challenging to link to breeding traits. The dependence on environments adds a further level of complexity. At the same time, basing crop growth models only on breeding traits and their combinations and describing within the model how these traits are altered by the environment is generally unfeasible. Even if all the mechanisms involving these traits and their response to growing conditions were well understood and amenable to inclusion in a model, the latter would have large parameter requirements. While these parameters would be better linked to “true traits,” the large amount of field measurements needed for a proper model parameterization would diminish its wide applicability.

A case in point is the RUE. The RUE is a central parameter in most crop growth models, but needs to be decomposed into its component traits that breeders can select for. The component traits behind RUE include the leaf photosynthetic capacity and the spatial distribution of this photosynthetic capacity over the canopy (Rodriguez et al., 1999). The latter is in turn affected by breeding traits such as leaf angle, leaf phenology, and the carbohydrate source-sink balance during the grain filling of cereals (Reynolds et al., 2000)—some of which could be altered by the plant–plant interactions that are important in intercrops. Although automated phenotyping facilities will enable monitoring the component traits behind RUE in the future (Furbank et al., 2019), their potential use in crop growth models to assist breeding can only be realized if the corresponding field assessments are performed in real intercrops to accommodate the physiological and biochemical mechanisms that are specific for the beneficial effects of intercrops. Keeping in mind the high degree of complexity in the existing comprehensive crop growth models such as APSIM and STICS, incorporating both some additional component traits behind RUE and the interspecific plant–plant interaction mechanisms required to truly simulate intercrops for breeding purposes, is perhaps unfeasible.

A more promising approach is developing dedicated minimalist crop growth models incorporating the plant characteristics and traits that are particularly important for the outcome of the intercrop. While even in these models some parameters cannot be immediately linked to traits for breeding, most of these minimalist crop growth models include parameters that either are true breeding traits important in an intercrop context, or could be easily linked to them; e.g., specific leaf area and plant height (Gou et al., 2017; Berghuijs et al., 2020; Tan et al., 2020). These are good candidates to be included in models to assist breeders. For example, plant height has been selected by breeders during several decades of cereal improvement and could be justified as a trait of interest also in the modeling.

A further aspect to consider when assessing the potential of crop growth models in intercropping is the ultimate goal of the intercrop, and how much such performance is affected by specific crop traits. For example, if maximizing the total intercrop community (seed) yield is the most important goal, then crop height of the component species might be less important. But if instead the individual yields of the component species matter most, then the crop height of the individual intercrop components is an important trait to consider (Berghuijs et al., 2020).

In summary, for models to be effective in assisting breeding of intercrops, they need to be designed so that they can be used to predict the best trait combinations for the specific end-use of the intercrop. To this end, models need (i) to incorporate the relevant plant features and mechanisms driving interspecific plant–plant interaction in the model; (ii) rely on parameters that are closely linked to the traits that breeders would select for; and (iii) be calibrated and validated with field data that are assessed in intercrops, if possible using advanced field phenotyping technologies to fulfil the parameter requirements of the common crop growth models. Due to their lower complexity and much reduced parameter requirement, minimalist crop growth models are more likely to incorporate the above elements than comprehensive and parameter-rich crop growth models such as APSIM and STICS.

## Author Contributions

MW: conceptualization. MW and DR: writing—original draft preparation. DR, EA, and GV: writing—review and editing. DR, EA, GV, and MW: project administration and funding acquisition. All authors have read and agreed to the published version of the manuscript.

## Funding

This research was funded by the DIVERSify project, a grant from the European Union’s Horizon 2020 Research and Innovation Programme under grant agreement No. 727284. GV and MW were also supported by a grant from the Swedish Research Council Formas (Grant Number 2018-02321).

## Conflict of Interest

EA is employed by Saatzucht Gleisdorf GmbH.

The remaining authors declare that the research was conducted in the absence of any commercial or financial relationships that could be construed as a potential conflict of interest.

## Publisher’s Note

All claims expressed in this article are solely those of the authors and do not necessarily represent those of their affiliated organizations, or those of the publisher, the editors and the reviewers. Any product that may be evaluated in this article, or claim that may be made by its manufacturer, is not guaranteed or endorsed by the publisher.
